# Relationship Between Diabetes Mellitus and Periodontal/Peri-Implant Disease: A Contemporaneous Review

**DOI:** 10.1016/j.identj.2024.03.010

**Published:** 2024-04-12

**Authors:** Shabnam Enteghad, Farinaz Shirban, Mohammad Hossein Nikbakht, Mohammad Bagherniya, Amirhossein Sahebkar

**Affiliations:** a School of Dentistry, Shiraz University of Medical Sciences, Shiraz, Iran; b Department of Orthodontics, Dental Research Center, Dental Research Institute, School of Dentistry, Isfahan University of Medical Sciences, Isfahan, Iran; c School of Dentistry, Isfahan University of Medical Sciences, Isfahan, Iran; d Food Security Research Center, Isfahan University of Medical Sciences, Isfahan, Iran; e Anesthesia and Critical Care Research Center, Isfahan University of Medical Sciences, Isfahan, Iran; f Department of Community Nutrition, School of Nutrition and Food Science, Isfahan University of Medical Sciences, Isfahan, Iran; g Biotechnology Research Center, Pharmaceutical Technology Institute, Mashhad University of Medical Sciences, Mashhad, Iran; h Applied Biomedical Research Center, Mashhad University of Medical Sciences, Mashhad, Iran

**Keywords:** Diabetes mellitus, Periodontal disease, Peri-implant disease, Review

## Abstract

The prevalence of diabetes mellitus (DM), a major chronic disease and a leading cause of death and disability around the world, is rising. According to the latest data, the global prevalence of DM has increased to 463 million (9.3% of adults) in 2019 and is estimated to reach 700 million by 2045. Periodontal disease, result of periodontium inflammation, is a common, chronic disease and has long been considered one of the complications of DM. Moreover, literature reflects a spectrum of conflicting viewpoints on the effect of diabetic conditions on the implant treatment strategies. The current review aims to update the recent epidemiologic evidence regarding the relationship between DM and periodontal/peri-implant disease, emphasising the effects of glycaemic control on the severity of these diseases and describing the pathobiological mechanisms underlying this association. This review's findings indicate a bidirectional relationship between DM and periodontal/peri-implant disease and that this relationship seems causal, implying that controlling these two diseases might help prevent each other's incidence. Additionally, the severity of periodontal/peri-implant disease is directly related to metabolic control. Although patients with diabetes can obtain implant success similar to those in systemically healthy individuals, an increased risk of peri-implantitis has been reported in DM patients. Therefore, the importance of glycaemic control and maintaining proper oral hygiene cannot be overstated.

## Introduction

Diabetes mellitus (DM) is a collective term for a set of metabolic disorders, with chronic hyperglycaemia being the primary manifestation.^1^ The aetiology of DM is either related to autoimmune destruction of β-cell, usually leading to absolute insulin deficiency (type 1 diabetes), or a progressive loss of sufficient β-cell insulin secretion, usually on the background of insulin resistance (type 2 diabetes).^2^ According to the latest data from the International Diabetes Federation, the global prevalence of DM reached 529 million with and age-standardised prevalence of 6.1% in 2021, and is estimated to reach 1.31 billion by 2050.^3^ Periodontal diseases (PD) periodontium infection, are prevalent, affecting almost 20% to 50% of the world population in both developed and developing countries.^4^ Based on the Global Burden of Disease Study (2016), severe PD is ranked as the 11th most prevalent condition worldwide.^5^ The high prevalence of PD in different age groups makes it a public health concern. Strong evidence shows the link between PD and systemic diseases such as diabetes, cardiovascular disease, and undesirable pregnancy outcomes.^4^ Both diabetes and PD are chronic, inflammation-related diseases, sharing the same risk factors and negatively affecting each other.^6^ That can explain why PD has been considered the sixth complication of diabetes.^7^

It is common for patients with diabetes to have multiple missing teeth due to PD or other causes. Therefore, replacing missing teeth in these patients using dental implants is one of the most common treatment plans. However, there is evidence to suggest that implant treatment must be chosen with caution in patients with diabetes due to the higher susceptibility of these patients to complications such as peri-implant diseases.^8^ Despite the tremendous progress and high success rate of implant treatment, which has enhanced both the patients’ quality of life and job satisfaction for dentists, complications due to peri-implant diseases are prevalent.^9^^,^ ^10^ Peri-implant diseases are classified into peri‐implant mucositis and peri‐implantitis. Peri-implant mucositis refers to bleeding on probing and visible signs of inflammation caused by plaque. Peri‐implantitis is a plaque‐related pathologic state marked by inflammation in the peri‐implant mucosa and progressive bone loss around the implant.^11^ According to 2 meta-analyses, the mean subject-based prevalence of peri-implant mucositis and peri-implantitis is approximately 45% and 20%, respectively.^12^^,^ ^13^ It has long been suggested that in patients with metabolic diseases such as DM, implant treatment outcomes might be affected due to impaired wound healing, the prevalence of microvascular disease, and dysregulated host response. Although diabetes is generally considered a relative contraindication for implant treatment, well-managed individuals with diabetes may be suitable candidates for implant therapy, whereas patients with diabetes with poorly regulated glycaemic conditions may not receive the advantages of implant therapy. Currently, the topic is a source of ongoing debate, with scholars presenting contrasting arguments in the literature.^14^, ^15^, ^16^, ^17^, ^18^, ^19^

The connection between diabetes and periodontal/peri-implant diseases is complex, and there is considerable heterogeneity in the clinical evidence on the association between these diseases. Furthermore, the mechanisms underlying the relationship between these 2 conditions are not entirely understood. Therefore, this review aims to update the current evidence of the relationship between DM and periodontal or peri-implant diseases. This article reviews the epidemiological studies on the role of DM as a risk factor for periodontal and peri-implant diseases, emphasizing the effects of glycaemic control on the severity of these diseases and describes the pathobiological mechanisms involved in the association between DM and periodontal/peri-implant diseases.

## Methods

The literature search was conducted on PubMed, Web of Science, Scopus, and Google Scholar data bases up to January 2024 using the following search terms: diabetes mellitus OR hyperglycaemia OR glycaemic control OR periodontal disease OR periodontitis OR gingivitis OR peri-implant disease OR peri-implantitis OR peri-implant mucositis OR tooth loss OR implant survival. The comprehensive literature search was executed collaboratively by Sh E and F Sh, ensuring a thorough exploration of relevant studies. The filters used were humans and the English language. To avoid bias due to the use of duplicate records, when one systematic review was included, the previously published original articles on the same subject as the systematic review were excluded.

## Results

The main characteristics of the reviewed studies, including type of study, aims, and main findings, are summarised in the tables in this article. The summarised information from systematic reviews and meta-analyses is presented in Table 1, and the data extracted from original studies is shown in Table 2. Here, the main findings of this review are categorised into epidemiological/clinical evidence and biological mechanisms, as discussed below.

**Table 1:** The association between diabetes mellitus and periodontal/peri-implant diseases based on systematic reviews and meta-analyses.

Author/Year	Study design	Aim & Objective	Number of included studies	Main outcomes
Type 1 diabetes mellitus (T1DM)				
Ismail et al.^49^ 2015	Systematic review	To evaluate the oral health status of T1DM children	37	T1DM children have poorer periodontal health status as well as higher plaque accumulation compared with healthy children. However, there were no significant differences in periodontal parameters over time, according to 3 cohort studies.
Dicembrini et al.^20^ 2020	Systematic review and meta-analysis	1. To evaluate the prevalence and severity of periodontal disease (PD) in T1DM patients compared to the general population 2. To investigate the relationship between severity of PD and glycaemic control in T1DM patients.	19	The PD prevalence in T1DM patients was 18.5%; 95% CI [8.0, 37.1]. Compared with healthy population, T1DM patients have more than a doubled chance for having PD (MH-OR 2.51; 95% CI [1.32, 4.76]). T1DM patients have 0.506 mm higher clinical attachment loss (CAL) than healthy controls. Among T1DM patients, those with suboptimal glycaemic control (HbA1c > 7%) are affected by a more severe form of PD, reflected in a 0.71 mm greater CAL depth than those with good glycaemic control.
Zainal Abidin et al.^33^ 2021	Systematic review and meta-analysis	To evaluate the periodontal health status of children and adolescents with T1DM	11	Compared to non-DM children, DM children had significantly higher mean values for plaque index (PI), gingival index (GI), CAL and PPD based on the Standardised Mean difference (SMD) and 95% confidence intervals (CI): PI (SMD 0.54; 95% CI [0.20, 0.87]); GI (SMD 0.63; 95% CI [0.39, 0.87]); CAL (SMD 0.79; 95% CI [0.52, 1.05]); PPD (SMD 0.67; 95% CI [0.23, 1.11])
Jensen et al.^34^ 2021	Systematic review and meta-analysis	To evaluate the severity of PD risk markers in children and adolescents with T1DM	23	Random effects meta-analyses showed that compared to healthy controls, children and adolescents with T1DM had higher values of risk markers for PD including: PI (SMD 0.45 (95% CI [0.21, 0.70]), GI (SMD 0.51; 95% CI [0.28, 0.74]), bleeding on probing (BOP) (SMD 0.61; 95% CI [0.40, 0.82]), PPD (SMD 0.55; 95% CI [0.22, 0.87]) and CAL (SMD 0.54; 95% CI [0.29, 0.78]) (all *P* < .001).
Type 2 diabetes mellitus (T2DM)				
Schimmel^54^ 2018	Systematic review and meta-analysis	To investigate the influence of systemic diseases on implant survival in geriatric patients (≥75 y)	7	In T2DM patients, rates of implant survival were reported to be between 86.3% (2-y observation period) and 100% (1-y observation period). Implant survival rate might be affected by poor glycaemic control (HbA1c ≥ 8.0%).
Lagunov et al.^43^ 2019	Meta-analysis	To evaluate BOP, PPD, and marginal bone loss (MBL) in T2DM and control patients	7	There was a statistically significant difference between patients with controlled T2DM (HbA1c 6.1%‐8%) and healthy group in terms of implant parameters such as MBL, BOP, and PPD (mean difference: 0.10 mm, 9%, and 0.37 mm, respectively). T2DM patients had a higher risk of peri‐implant disease, although diabetes was controlled in these patients.
Singh^56^ 2019	Systematic review	To investigate the effect of implant placement techniques on the implant survival rate, in well-controlled T2DM patients	14	Published studies reported no difference in dental implant survival rate among well‑controlled, moderately, and poorly controlled DM patients and non-DM individuals. Implant survival rate is similar in conventional and flapless techniques (94.2% and 92.3%, respectively).
Wu et al.^21^ 2020	Systematic review and meta-analysis	To review the epidemiologic association between T2DM and periodontitis.	53	The Adjusted prevalence of periodontitis was significantly higher in T2DM patients (OR 1.58; 95% CI [1.38, 1.81]), and vice versa (OR 4.04; 95% CI [2.48, 6.59]). Periodontal status in T2DM patients was significantly worse, as shown by a 0.61 mm higher pocket probing depth (PPD), a 0.89 mm CAL and about 2 more missing teeth (all *P* = .000), compared to non-DM patients. Based on the results of the cohort studies, the risk of developing periodontitis is elevated by 34% in T2DM subjects (RR 1.34; 95% CI [1.11, 1.61]). Although both severe and mild periodontitis elevated the risk for incident T2DM (RR 1.53, 95% CI 1.27, 1.83 and RR 1.28, 95% CI [1.07, 1.54], respectively), mild periodontitis had a less robust impact on the incidence of T2DM.
Shang et al,^41^ 2021	Systematic review and meta-analysis	To assess the influence of hyperglycaemia on dental implant treatment	9	Implant failure rates and PPD were not significantly different between DM and non-DM patients. BOP and peri-implant bone loss (PIBL) were significantly higher among DM patients. Peri-implant parameters (BOP, PPD, and PIBL) were not associated with glycaemic level among T2DM patients with good oral hygiene maintenance.
Tan^42^ 2021	Systematic review and meta-analysis	To assess implant survival and possible dose-response relationship between HbA1c and peri-implant parameters in T2DM patients with different glycaemic control.	22	T2DM patients with HbA1c<8% had a high implant survival rate of 92.6%-100% in the first 3 y. In patients with HbA1c>8%, osseointegration was delayed. Increase in HbA1c level was associated with worsening of clinical parameters. Each 1% increase in HbA1c was significantly associated with 10% higher BOP and 0.05 mm more MBL, but no association between glycaemic control and PPD was found.
Chopra et al.^65^ 2022	Systematic review	To investigate effects of inflamed periodontal tissues and periodontal pathogens on modulating AGE levels in individuals with or without T2DM and its influence on the glycaemic status	13	Individuals with T2DM and periodontitis had significantly greater levels of AGE (Advanced Glycation Endproducts) in their gingival crevicular fluid (GCF) (521.9 pg/mL) compared to healthy individuals with periodontitis (234.84 pg/mL). Tannerella forsythia was detected in the gingival tissues.
Thomas et al.^66^ 2023	Systematic review	1. To evaluate the levels of AGEs and RAGEs (AGE receptors) expression in patients with diabetes periodontitis and patients without diabetes periodontitis, and 2. To determine the correlation between AGEs and RAGEs levels and the severity of periodontal disease.	16	Patients with diabetes and chronic periodontal disease had a higher amount of AGEs compared to the patients without diabetes. In addition, the levels of AGEs and RAGEs are linked to CAL and PPD.
T1DM and T2DM				
Chrcanovic et al.^16^ 2014	Systematic review and meta-analysis	To evaluate the effects of DM on implant failure rates, MBL, and postoperative infections	14	A significantly higher MBL was observed in patients with diabetes compared with individuals without diabetes. The risk of implant failure did not differ between patients with diabetes and individuals without diabetes (RR 1.07; 95% CI [0.80, 1.44]).
Moraschini et al.^47^ 2016	Systematic review and meta-analysis	To assess whether there is any difference in implant failure rate or MBL between T1DM or T2DM subjects and non-DM subjects.	14	Risk of implant failure was not statistically different between T2DM subjects and non-DM subjects (RR 1.43; 95% CI [0.54, 3.82]). Similarly, the risk of implant failure was not statistically different between T1DM subjects and non-DM subjects (RR 3.65; 95% CI [0.33, 40.52]). The risk of implant failure did not differ between the two types of DM subjects (RR of 1.56; 95% CI [0.62, 3.91]) Patients with diabetes had a significantly higher MBL of 0.18 mm, compared to non-DM patients.
Naujokat et al.^55^ 2016	Systematic review	To assess postoperative complications, peri-implantitis, and implant failure rate in DM and non-DM patients	22	Poorly controlled DM is connected to impaired osseointegration, higher risk for peri-implantitis, and higher implant failure rate. Antibiotic and chlorhexidine administration appears to enhance implant success. Similar to healthy patients, implant treatment can be safely done in well-controlled DM patients.
Monje et al.^129^ 2017	Systematic review and meta-analysis	To investigate the association between DM and peri-implant diseases (peri-implant mucositis and peri-implantitis).	12	Meta-analyses calculated about 50% higher risk and about 90% higher odds of peri-implantitis in DM patients compared with non-DM subjects (RR 1.46; 95% CI [1.21, 1.77] and OR 1.89; 95% CI [1.31, 2.46]). In contrast, there was no statistically significant association between DM and peri-implant mucositis (RR = 0.92; 95% CI [0.72, 1.16] and OR = 1.06; 95% CI [0.84, 1.27]).
Ziukaite et al.^130^ 2018	Systematic review and meta-analysis	To assess the prevalence and odds of having DM in periodontitis patients	29	Diabetes prevalence was higher among subjects with periodontitis compared with subjects without periodontitis (12.8% and 9.4% respectively). The overall OR for DM patients to be among periodontitis patients relative to nonperiodontitis subjects was 2.59 (95% CI [2.12, 3.15]). The DM prevalence in periodontitis patients was underestimated when diabetes was self-reported compared with clinical assessment of the condition (6.5% and 17.3%, respectively). The prevalence of DM among periodontitis subjects was the highest in studies conducted in Asian countries (17.2%) and the lowest in those describing European populations (4.3%).
de Oliveira-Neto et al.^44^ 2019	Overview of systematic reviews	To evaluate the methodological quality of systematic review studies on dental implant in DM patients.	8	Meta-analysis of 2 high methodological quality studies showed that DM does not have an effect on survival rate of dental implants. However, marginal bone loss is significantly affected by DM.
Jiang et al.^40^ 2021	Systematic review and meta-analysis	To investigate the possible relationship between DM and complications of dental implants	10	Compared with healthy subjects, DM patients showed significantly higher complications such as MBL, PPD and bleeding around dental implants (MD: 0.10 mm, 0.30 mm, and 22.62%, respectively). Subgroup analysis based on HbA1c level and loading times found that while bleeding around implants was correlated with HbA1c levels, PPD was not associated with HbA1c level, but with the immediate loading of implant.
Weijdijk et al.^38^ 2021	Systematic review and meta-analysis	To assess the risk of tooth loss among patients with DM compared with non-DM individuals.	10	DM patients have a significantly higher risk of losing teeth (RR 1.63; 95% CI [1.33, 2.00]). The risk of tooth loss in DM patients was even higher when only T2DM patients or cross-sectional studies were considered. The risk of tooth loss was significantly increased in patients with poor glycaemic control. The studies originating from Asia and South America reported numerically greater risks of tooth loss.
Stöhr et al.^22^ 2021	Systematic review and meta‑analysis	To summarise the existing evidence on the bidirectional relationship between PD and DM	15	The summary relative risk (SRR) for DM incidence in periodontitis patients was 1.26; 95% CI [1.12, 1.41], compared to those without periodontitis. The SRR for periodontitis incidence in DM patients was 1.24; 95% CI [1.13, 1.37], compared to those without DM. The results show a positive bidirectional prospective association between PD and DM.
Dhingra et al.^32^ 2023	Meta-analysis	To investigate effect of periodontal treatment on the glycaemic control in periodontitis patients with T1DM and T2DM.	33	Periodontal treatment (subgingival intervention) resulted in a significant decrease in HbA1c levels. The reduction was 0.43% after 3-4 mo, 0.30% at 6 mo, and 0.50% at 12 mo, as compared to standard care or no treatment. Treating periodontitis with subgingival instrumentation enhanced the the glycaemic control in individuals with diabetes.
DM type: not reported				
Nascimento et al.^23^ 2018	Systematic review and meta‑analysis	To evaluate the association between poorly controlled diabetes and incidence or progression of periodontitis.	13	The risk for incidence or progression of periodontitis was elevated by 86% in DM patients (RR 1.86; 95% [CI 1.3, 2.8]). However, the information on the relationship between DM and periodontal destruction is scarce.
Aghaloo et al.^51^ 2019	Systematic review and meta-analysis	To assess the impact of systemic disorders including DM and osteoporosis on implant osseointegration	20	In the included studies, no differences were observed between 3-mo implant osseointegration in patients with and without DM. Implant survival rate was 98% (95% CI [96%, 99%]) in DM patients. However, diabetes might affect marginal bone loss and implant success in long term.
Meza Maurício et al.^52^ 2019	Umbrella review of systematic reviews	To summarise the evidence on DM association with peri-implant complications	12	Considering implant failure rate, three meta-analyses reported no statistically significant differences between DM and non-DM subjects. Evidence shows high levels of implant survival (83.5%- 100%), and low levels of implant failure (0%-14.3%) placed in DM patients. However, the impact of hyperglycaemia on implant survival and failure is still unknown. DM/hyperglycaemia appears to increase the risk of peri-implantitis.
Alwithanani^24^ 2023	Systematic review and meta-analysis	To assess the association of poorly managed DM with the development or progression of periodontitis	13	Meta-analyses of adjusted data indicated that DM increases the probability of developing or advancing to periodontitis by 86% (RR 1.86; 95% CI 1.3-2.8). Individuals with diabetes are more susceptible to acquiring periodontitis.
Li et al.^31^ 2023	Meta-analysis	To find the correlation between DM and peri-implant diseases in patients with osseointegrated dental implants	21	The study did not find a significant correlation between DM and peri-implant mucositis (OR: 0.739, 95% CI: 0.394- 1.383, *P* = .344). The findings indicated that individuals with DM had a greater likelihood of developing peri-implantitis compared to individuals without DM (OR: 1.553, 95% CI: 1.084-2.226, *P* = .016).
Dioguardi et al.^50^ 2023	Systematic review	To compare the status of peri-implanitistis parameters, between DM patients and non-DM individuals	11	Elevated BOP and MBL in DM patients than in non-DM subjects
Baniulyte et al.^131^ 2023	Meta-analysis	To compare the risk of peri-implanitistis between DM patients (and smokers) and non-DM individuals (and nonsmokers)	21	Elevated risk of peri-implantitis in DM patients and in smokers when compared to non-DM subjects and non-smokers.

**Table 2:** The association between diabetes mellitus and periodontal/peri-implant diseases based on observational studies.

Author/Year	Type of the study	Area	Aim & Objective	Number of subjects	Main outcomes
T1DM					
Sannino et al.^48^ 2020	Cohort	Italy	To evaluate implant treatment in partially edentulous patients with T1DM	53 T1DM 53 healthy Control	The difference between the implant survival rate of T1DM group and healthy controls was not significant (95.19% and 97.03%, respectively). Occurrence of infections, and MBL was not significantly different between the two groups. Implant treatment for partially edentulous patients can be a safe and predictable procedure for T1DM patients, as long as glycaemic levels are controlled and oral hygiene is maintained.
Chakraborty et al.^26^ 2021	Cross-sectional	India	To assess the possible impact of pubertal status and metabolic derangement on periodontal status of T1DM patients	110 T1DM 52 healthy siblings of similar age	PD, not just gingivitis, was significantly higher in children with diabetes (76.36%) compared with the controls (53.8%) Irrespective of pubertal status, T1DM children had higher GI, PI, BOP, and PPD than non-DM controls, although they had better oral hygiene. PD was associated with age, pubertal stage, and HbA1c, but not with the diabetes duration.
Jensen et al.^111^ 2021	Cross-sectional	Australia	1. To investigate the association between PD and glycaemic control in T1DM children 2. To evaluate oral microbiota of these patients in terms of diversity and composition	77 T1DM subjects	Early markers of PD was observed in 49% of patients. Adjusted for confounders, a significant positive correlation was found between glycaemic level and periodontal risk markers such as PI, GI, BOP and PPD >3 mm. With each 1% increase in HbA1c level, a 25% rise in BOP and a 54% rise in the rate of sites with PPD >3 mm was observed. HbA1c was positively correlated with increased richness and complexity of the plaque microbiome.
T2DM					
Quadri et al.^25^ 2020	Case-control	Saudi Arabia	To investigate the correlation of uncontrolled T2DM with periodontal status	166 periodontitis 332 controls	One of the most important predictors of periodontitis in Saudi Arabian adults is uncontrolled T2DM, which can increase the odds of having periodontitis by approximately 3 times (OR: 2.779; 95% CI [1.425-5.419]).
Alshahrani et al.^46^ 2020	Retrospective cohort	Saudi Arabia	To assess the clinicoradiographic status of narrow-diameter implants (NDIs) placed in prediabetic, T2DM, and non-DM patients.	20 prediabetes (HbA1c 5.5 to 6.4%) 22 poorly controlled T2DM (HbA1c ≥ 6.5%) 20 well-controlled T2DM (HbA1c < 5.7%) 20 self-reported non-DM (HbA1c level ≤ 5.7%)	Peri-implant PI, GI, PD, and mesiodistal crestal bone loss (CBL) levels were significantly higher in patients with pre-diabetes and poorly controlled T2DM than patients with well-controlled T2DM and non-DM controls. Poorly controlled T2DM patients had significantly higher peri-implant PI, GI, PD, and mesiodistal CBL levels than prediabetes patients.
Alqahtani et al.^132^ 2020	Cross-sectional	Saudi Arabia	To investigate the implant survival in cigarette-smokers (CS) and never-smokers (NS) with T2DM.	25 CS + T2DM 26 CS 25 NS + T2DM 25 NS	The mean HbA1c levels were significantly higher among T2DM patients than individuals without T2DM, irrespective of smoking status. Peri-implant PI, PPD, and CBL were significantly higher among CS+T2DM and NS+T2DM and CS compared to NS. T2DM patients had considerably greater peri-implant BOP than those without T2DM. In T2DM patients, chronic hyperglycaemia is probably a more important mediator of inflammation compared to cigarette smoking.
Hasan et al.^133^ 2021	Cohort	Bangladesh	To elucidate the correlation of self-care and oral hygiene practices to PD in DM patients.	379 T2DM	There was a high prevalence of any (CPI code 2+3+4: 75.7%) and severe form (CPI code 4: 35.1%) of PD in T2DM patients. Poor glycaemic control led to 2.7 times increased odds of PD, while commitment to a healthy diet, physical activity, and good oral hygiene decreased the odds of PD by 64%, 85% and 92%, respectively. HbA1c>7%, diabetes duration >5 y, hypertension, and high BMI were significantly correlated with the diagnosis of severe PD.
Ge et al.^27^ 2021	Cross-sectional	china	To assess the association between the DM control and periodontitis	3064 T2DM	Among T2DM subjects, the prevalence of moderate and severe periodontitis was 10.57%. The proportion of poorly-controlled T2DM patients was significantly higher in severe periodontitis group than non-severe periodontitis group (68.52% and 60.99%, respectively). Glycaemic control was positively correlated with severe periodontitis (OR 2.8, *P* < .05).
Stoicescu et al.^35^ 2021	Cross-sectional	Romania	To investigate the association between glycaemic control and periodontal parameters in T2DM patients with generalised chronic periodontitis.	182 T2DM	Patients with uncontrolled diabetes (HbA1c ≥7%) had significantly higher mean values for full‑mouth plaque accumulation, PPD, CAL, the number of sites with PD ≥5 mm, and lower number of remaining teeth, compared with patients with HbA1c <7%.
Lorean et al.^45^ 2021	Retrospective cohort	Romania	To assess implant survival rates, MBL, and the influence of prosthesis type among T2DM patients, with high HbA1c values	25 moderately controlled T2DM patients (HbA1c: 6.9% to 8.0%) 13 poorly controlled T2DM patients (HbA1c: 8.1% to 10.0%)	The overall survival rate of implants was 98.4%. In both groups, higher bone loss was observed in the maxilla than in the mandible. Patients with poor glycaemic control had a significantly higher MBL (2.33±2.85 mm) than those with moderately controlled diabetes (1.86±2.21 mm). Greater bone loss rates was observed in the group of patients using removable prostheses compared with those using fixed prostheses.
Takeda et al.^36^ 2021	Cross-sectional	Japan	To evaluate the association of PD inflammatory parameters with DM an obesity	71 T2DM	In T2DM patients, periodontal inflamed surface area (PISA) was significantly associated with FPG (>175 mg/dL) and HbA1c, but not with obesity parameters, controlled for confounders like full-mouth plaque control.
Romano et al.^37^ 2021	Cross-sectional	Italy	To assess the periodontal health status and its association with glycaemic control in T2DM patients	104 T2DM	Predictors of severe periodontitis were family history of T2DM, poor diabetic control (HbA1c ≥ 7%), and levels of C-reactive protein. Poor glycaemic control was linked to severe periodontitis, waist circumference, an imbalanced diet, and a sedentary lifestyle. 1% rise in HbA1c level resulted in 89.6 mm^2^ increase in PISA. 10 mm^2^ rise in PISA resulted in 2% higher odds for having HbA1c ≥ 7%. Periodontitis and poor glycaemic control are bidirectionally associated with each other.
Yu et al.^114^ 2022	Case-control	Korea	To analyse the effect of DM on intragingival microbial profile of periodontitis patients	39 T2DM	DM impact the advancement of periodontitis by enhancing the bacterial network within the gingival tissue
Arshad et al.^100^ 2022	Case-control	Pakistan	To assess relation between levels of matrix metalloproteinase-9 (MMP-9) and T2DM in chronic periodontitis patients	30 T2DM	The levels of MMP-9 in DM patients with chronic periodontitis were nearly doubled (156.95 ± 29.80 ng/mL) compared to the values in the control group (74.96 ± 6.32 ng/mL) (*P* < .001). Periodontal parameters were significantly more severe in DM individuals and chronic periodontitis in comparison to the control group.
Pandian et al.^115^ 2023	Case-control	India	To evaluate the diversity and prevalence of periodontal pathogens found in the subgingival plaque of individuals with severe chronic periodontitis, with and without diabetes	56 T2DM	Greater bacterial burden in the DM group, *T*. forsythia (*P* < .037) and *T. denticola* (*P* < .003), compared to the non-DM group
Todescan et al.^28^ 2023	Cross-sectional	Canada	To ascertain the frequency of periodontitis in children and adolescents diagnosed with T2DM To investigate if inadequate glycaemic control is linked to a higher incidence of the periodontitis.	121 T2DM	The study confirmed a significant correlation between the occurrence of periodontitis and glycosylated haemoglobin (HbA1c) based on both bivariate (odds ratio [OR] 1.31 [95% CI, 1.13-1.53], *P* = .001) and multivariate (OR, 1.29 [95% CI, 1.03-1.61], *P* = .03) analyses. The prevalence of PD is affected by uncontrolled HbA1c levels.
Rahim et al.^29^ 2023	Cross-sectional	Pakistan	To investigate the correlation and intensity of periodontal clinical parameters and oral hygiene with HbA1c levels in individuals without diabetes and those with T2DM.	96 T2DM	According to the Oral Hygiene Index-Simplified (OHI-S); individuals with uncontrolled T2DM had the highest prevalence of poor oral hygiene, with 29 individuals (20.1%) exhibiting this condition. This was followed by controlled T2DM patients, with 22 individuals (15.3%), and non-DM individuals, with 14 individuals (9.7%) (*P* = .03). The periodontal and oral hygiene status of uncontrolled T2DM patients were shown to be worse than those of non-DM participants and controlled T2DM patients.
T1DM and T2DM					
Raedel et al.^39^ 2021	Cross-sectional	Germany	To evaluate the influence of DM on the periodontal treatment outcomes in terms of tooth loss	4139 T1DM 22,430 T2DM+medication 23,576 T2DM 365,573 control	T1DM and T2DM appear to increase the risk of losing teeth after periodontal treatment. The 4 y survival rates (no extraction) in the T1DM, T2DM+medication, and T2DM groups were 51.7%, 54.0% and 57.7%, respectively. The control group had a considerably greater survival rate of 65.9% (*P* < .0001).
DM type: not reported					
French et al.^53^ 2021	Cohort	Canada	To evaluate the clinical performance of dental implants in long-term	10,871 dental implants in 4248 patients	A significantly positive correlation was found between DM and implant failure (HR 2.25 95% CI [1.04, 4.89]).
Rekawek et al.^134^ 2021	Retrospective cohort	USA	To assess the protective effect of hygiene visits on the development of peri-implantitis in DM patients.	748 implants in 286 patients	Patients with DM were 49% more likely to develop peri-implantitis (hazard ratio (HR) 1.491; 95% CI [0.758, 2.936], *P* = .2475) than healthy individuals. The risk peri-implantitis incidence was reduced by 20% with each hygiene visit (HR 0.805; 95% Cl [0.394, 1.647], *P* = .5528).
Park et al.^30^	Cohort	Korea	To examine whether the occurrence of diabetes is influenced by the recovery from or development of PD	111,611 subjects	The individuals who successfully recovered from PD exhibited a decreased risk of developing diabetes compared to those who consistently had PD (adjusted hazard ratio (HR) 0.930, 95% confidence interval 0.865-1.000, *P* = .050). Conversely, those who developed PD had an elevated risk of developing diabetes compared to those who remained free of PD. The longitudinal of PD status is linked to an increased chance of developing diabetes.

### Epidemiological/clinical evidence

#### The association between DM and periodontal/peri-implant disease

Multiple systematic reviews, cross-sectional, case-control, and cohort studies have investigated the association between DM and periodontal/peri-implant diseases. In 2020, a systematic review and meta-analysis by Dicembrini et al. examined data from 22,172 individuals of various nationalities included in 5 epidemiological studies. The results of this study demonstrated that the prevalence of PD in type 1 DM (T1DM) was 18.5%, and the odds of having PD were more than doubled in children with T1DM, with Mantel-Haenszel odds ratios (MH-OR) of 2.5.^20^ In a study by Wu et al., a systematic review and meta-analysis involving 11,459 participants revealed a significant association between type 2 diabetes mellitus (T2DM) and periodontitis.^21^ The odds ratio (OR) for periodontitis in individuals with T2DM was calculated as 1.58 (*P* < .001). Additionally, the study found a substantially higher adjusted prevalence of T2DM among periodontitis patients, with an OR of 4.04. The authors concluded that T2DM could elevate the risk of developing periodontitis by 34% (*P* = 0.002). The study also explored the reverse association, discovering that severe periodontitis increased the risk of T2DM by 53% (*P* = 0.000), while mild periodontitis had a less pronounced effect (relative risk (RR) = 1.28, *P *= 0.007).

A 2021 systematic review of cohort studies^22^ reported a summary relative risk (SRR) of 1.26 for the incident DM in patients with periodontitis and an SRR of 1.24 for the incident of periodontitis in patients with DM. The lower risk of developing periodontitis in DM patients reported in this study and the study of Wu et al.^21^ compared to the study of Nascimento et al.^23^ could be because the impact of poorly controlled DM on the incidence of PD was evaluated in the latter. A systematic review and meta-analysis published in 2023 assessed the association of poorly managed DM with the development or progression of periodontitis. Results indicated that DM increases the probability of developing or advancing to periodontitis by 86% (RR 1.86; 95% CI 1.3-2.8). The study concluded that individuals with diabetes are more susceptible to acquiring periodontitis.^24^

A 2020 case-control study on a population of 498 Saudi Arabian adults demonstrated uncontrolled T2DM (HbA1c ≥7%) as an essential predictor for periodontitis and reported a nearly 3-fold increase in odds of being diagnosed with periodontitis compared to patients without diabetes.^25^

Findings of a 2021 cross-sectional study on 162 participants revealed that PD, not only gingivitis, was significantly higher in T1DM (76.36%) than the healthy control group (53.8%).^26^ Furthermore, two another 2021 cross-sectional studies investigated the correlation of PD with glycaemic control in patients with diabetes.^26^^,^ ^27^ Chakraborty et al. reported that in T1DM patients, PD was significantly correlated with glycaemic control, manifested by the HbA1c level (OR = 1.5).^26^ Ge et al. also reported a significant correlation between diabetes glycaemic control and severe periodontitis (OR = 2.8).^27^

In a recent cross-sectional study, the frequency of periodontitis in children and adolescents diagnosed with T2DM was evaluated. In addition, the study aimed to investigate if inadequate glycaemic control is linked to a higher incidence of the periodontitis. The study found a significant correlation between the occurrence of periodontitis and glycosylated haemoglobin (HbA1c) based on both bivariate (odds ratio [OR] 1.31 [95% CI, 1.13-1.53], *P* = 0.001) and multivariate (OR, 1.29 [95% CI, 1.03-1.61], *P* = .03) analyses. Results showed that the prevalence of PD is affected by uncontrolled HbA1c levels.^28^ In another recent cross-sectional design, researcher investigated the correlation and intensity of periodontal clinical parameters and oral hygiene with HbA1c levels in individuals without diabetes and those with T2DM. Results revealed that individuals with uncontrolled T2DM had the highest prevalence of poor oral hygiene, with 29 individuals (20.1%) exhibiting this condition. This was followed by controlled T2DM patients, with 22 individuals (15.3%), and non-DM individuals, with 14 individuals (9.7%) (*P* = .03). In brief, the study showed that periodontal and oral hygiene status of uncontrolled T2DM patients were shown to be worse than those of non-DM participants and controlled T2DM patients.^29^ In a recent cohort study, researcher examined whether the occurrence of diabetes is influenced by the recovery from or development of PD. Findings demonstrated that the individuals who successfully recovered from PD exhibited a decreased risk of developing diabetes compared to those who consistently had PD (adjusted hazard ratio (HR) 0.930, 95% confidence interval 0.865-1.000, *P* = .050). Conversely, those who developed PD had an elevated risk of developing diabetes compared to those who remained free of PD. In conclusion, the longitudinal of PD status is linked to an increased chance of developing diabetes.^30^

Regarding the association between DM and peri-implant diseases, in a recent meta-analysis, researchers investigated the correlation between DM and peri-implant diseases in patients with osseointegrated dental implants. Results showed that although the study did not find a significant correlation between DM and peri-implant mucositis (OR: 0.739, 95% CI: 0.394-1.383, *P* = .344), findings confirmed that individuals with DM had a greater likelihood of developing peri-implantitis compared to individuals without DM (OR: 1.553, 95% CI: 1.084-2.226, *P* = .016).^31^ In another recent study, meta-analysis of 33 studies aimed to investigate effect of periodontal treatment on the glycaemic control in periodontitis patients with T1DM and T2DM. Researchers demonstrated that periodontal treatment (subgingival intervention) resulted in a significant decrease in HbA1c levels. The reduction was 0.43% after 3 to 4 months, 0.30% at 6 months, and 0.50% at 12 months, as compared to standard care or no treatment. Collectively, Treating periodontitis with subgingival instrumentation enhanced the the glycaemic control in individuals with diabetes.^32^ Recently, in 2023, Baniulyte et al. in a meta-analysis confirmed the findings of previous studies on the increased risk of peri-implantitis in DM patients compared to non-DM individuals .

Findings from the above-mentioned cross-sectional studies and systematic reviews demonstrate that there might be a causal relationship between DM and periodontal/peri-implant diseases, and this relationship seems to be bidirectional for DM and PD, indicating that managing these two diseases may help prevent the occurrence of the other.

#### Effects of diabetes mellitus on periodontal parameters

This review evaluated various outcome indicators, including clinical attachment loss (CAL), periodontal inflamed surface area (PISA), and tooth loss to assess the impact of DM on periodontal parameters.

##### Clinical attachment loss

Most studies evaluated CAL as a valid parameter for diagnosing and classifying PD. Several studies^20^^,^ ^21^^,^ ^33^^,^ ^34^ found significantly higher values of CAL depth in DM patients of both types compared to patients without diabetes. Results from a meta-analysis of studies on 18 cross-sectional studies, including 9571 participants, indicated that patients with T2DM had a 0.89 mm higher CAL depth than non-DM controls.^21^ Regarding T1DM, the mean CAL difference between T1DM subjects and controls was reported to be 0.506 mm in favour of children without diabetes according to another meta-analysis of 9 studies, including 3869 patients.^20^ Two random-effects meta-analyses reported a standardised mean difference of medium^34^ and high^33^ effect size for CAL in T1DM children compared with healthy controls. Furthermore, considering the glycaemic control, a meta-analysis of 3 studies including 210 subjects reported that in well-controlled T1DM patients, the CAL depth is 0.71 mm less than in patients with HbA1c > 7%.^20^ Similarly, higher CAL was reported in patients with poor glycaemic control than in well-controlled T2DM patients in a cross-sectional study of 182 patients.^35^

##### Periodontal inflamed surface area (PISA)

Using the periodontal inflamed surface area (PISA) index, 2 studies showed the link between periodontitis and DM. PISA is a periodontal parameter introduced for the measurement of periodontitis severity. It measures the amount of bleeding epithelium around an individual tooth in mm^2^. One study calculated PISA and periodontal epithelial surface area (PESA) in 71 T2DM patients. It was shown that PISA was significantly correlated with HbA1c, independent of confounders such as oral hygiene.^36^ Another study in 104 T2DM patients reported that a 1% rise in HbA1c level resulted in an 89.6 mm^2^ increase in PISA. In addition, a 10 mm^2^ rise in PISA resulted in 2% higher odds of having HbA1c ≥ 7%. This indicates a robust bidirectional relationship between periodontitis and inadequate glycaemic control^37^

##### Tooth loss

One meta-analysis reported a higher risk of tooth loss in patients with DM types 1 and 2 by 63%. This risk increased when only T2DM and cross-sectional studies were considered.^38^ However, this study did not distinguish the causes of tooth loss, which could be either caries or PD. Another meta-analysis stated that T2DM patients have approximately 2 more lost teeth than healthy individuals.^21^ In a study based on massive data from a German population, it was reported that the proportion of non-DM patients who had no extractions in 4 years after periodontal therapy was 65%. This proportion was significantly lower at 51.7% for the T1DM group, 54.0% for the T2DM+medication group, and 57.7% for the T2DM group.^39^ Comparing patients with good and poor glycaemic control, a higher number of lost teeth has been reported in poorly controlled diabetes (HbA1c>7).^35^

#### Effects of diabetes mellitus on peri-implant parameters

Regarding peri-implant parameters, effects of DM on marginal bone loss (MBL), pocket probing depth (PPD), bleeding on probing (BOP), and implant survival were evaluated.

##### Peri-implant marginal bone loss

Higher levels of peri-implant MBL in patients with diabetes than in patients without diabetes have been reported by many systematic reviews and observational studies.^40^, ^41^, ^42^, ^43^, ^44^, ^45^, ^46^ According to two meta-analyses, DM patients undergoing implant treatment exhibited approximately 0.2 mm more marginal bone loss than healthy patients.^16^^,^^47^ However, a cohort study by Sannino et al. found no difference in peri-implant MBL between well-controlled T1DM patients with good oral hygiene and healthy controls in a 2-year follow-up.^48^

A systematic review and meta-analysis of 22 studies published by Tan et al.^42^ assessed the dose-response relationship between implant clinical parameters and HbA1c levels in patients with T2DM. This study established a significant dose-response connection between glycaemic control and per-implant MBL (0.05 mm more bone loss per HbA1c category). This is while Shang et al. reported no association between peri-implant parameters, including MBL, and glycaemic level among patients with T2DM who maintained strict oral hygiene.^41^

##### Pocket probing depth

Results of 4 systematic reviews^21^^,^^33^^,^^34^^,^^49^ and one cross-sectional study^26^ included in the present review showed deeper periodontal pockets in DM patients compared to controls. According to the findings of a meta-analysis, the mean difference between PPD in T2DM patients and healthy controls was 0.61 mm.^21^ Two random-effects meta-analyses reported that the standardised mean difference for PPD between T1DM patients and healthy controls was medium effect size.^33^^,^^34^ Furthermore, among DM patients, higher pocket depth and a higher prevalence of areas with PPD > 5 mm were shown in poorly glycaemic-controlled patients compared with patients with reasonable glycaemic control, according to one cross-sectional study.^35^

Regarding the probing depth of dental implants, a systematic review and meta-analysis of 10 cohort studies with 625 participants reported higher PPD around implants in patients with diabetes than in non-DM patients.^40^ The same result was reported by another meta-analysis of prospective studies and one retrospective cohort study.^43^^,^^46^ However, another systematic review failed to find any significant differences regarding PD between DM and control.^41^ Regarding the effect of glycaemic control on probing depth, none of the included studies found any difference between different glycaemic control levels.^40^, ^41^, ^42^

##### Bleeding on probing

Among the reviewed studies, only one systematic review and one cross-sectional study evaluated the effect of DM on the BOP of natural teeth. Both studies reported higher BOP in T1DM than in non-DM patients.^26^^,^^34^ This is while five systematic reviews and meta-analyses on dental implants found higher BOP around implants in patients with diabetes compared to patients without diabetes.^40^, ^41^, ^42^, ^43^^,^^50^

Moreover, 2 of these studies stated that as the HbA1c level increases, the BOP of peri-implant tissues will also increase.^40^^,^^42^ According to Tan et al., each 1% increase in HbA1c was significantly associated with 10% higher BOP.^42^ However, another systematic review reported no significant difference between different glycaemic control levels regarding BOP around implants in patients with good oral hygiene.^41^ In 2023 study, Dioguardi et al. compared the status of peri-implanitistis parameters, between DM patients and non-DM individuals and found an elevated BOP and MBL in DM patients than in non-DM subjects.^50^

##### Implant survival

Most of the included studies in this review, including one umbrella review, 2 systematic reviews and meta-analyses, and one over-review of systematic reviews, reported that T2DM does not influence the survival rate of dental implants.^41^^,^^44^^,^^51^^,^^52^ Similarly, one cohort study on 106 patients found no difference in the implant survival rate of well-controlled T1DM patients and healthy controls in a 2-year follow-up.^48^ According to the reviewed studies, the survival rate of implants in DM patients has been reported to be between 83.5% and 100%. It appears that short-term osseointegration and survival of implants are promising among DM patients. One systematic review reported a 3-month implant survival rate of 98% in DM patients with no significant difference between patients with well-controlled and poorly-controlled disease.^51^ Another systematic review also provided a 92.6% to 100% survival rate for the first 3 years.^42^ However, one published cohort of 4247 patients with 10,871 implants with up to 22 years of follow-up concluded a positive correlation between DM and implant failure (HR = 2.25, *P*-value = .040).^53^ Since it has been widely reported that DM and hyperglycaemia can increase the risk of peri-implantitis and lead to bone loss, DM patients may be at risk of implant failure in the long term.

Furthermore, it must be noted that most studies on implant survival have been done in patients with well-controlled diabetes. Most studies have not assessed the effect of glycaemic level, which may explain why no difference in the implant survival rates has been found. Two systematic reviews stated that poor glycaemic control might be associated with reduced implant survival.^54^^,^ ^55^ In contrast, another systematic review reported no difference in dental implant survival rate among well, moderately, or poorly glycaemic-controlled patients with diabetes and individuals without diabetes.^56^ Further longitudinal studies with longer follow-ups are needed to evaluate the possible effect of diabetes and different glycaemic control levels on the risk of implant failure.

#### Predictive value of periodontal/peri-implant parameters for identification of diabetes mellitus

Three studies introduced prediction models using periodontal indicators to assess the risk of DM in patients. Talakey et al. used periodontal parameters such as missing teeth, percentage of sites with PPD ≥ 6 mm, and the average PPD and classified 62.4% of participants correctly regarding having DM. The authors reported that the addition of these clinical periodontal assessments enhanced the performance of the Finnish Diabetes Risk Score (FINDRISC) and Canadian Diabetes Risk Questionnaire (CANRISK) tools.^57^ One study used the periodontal parameter of the percentage of sites with CAL > 3 mm while considering risk factors like family history of DM, hypertension, and smoking. It could correctly classify 69.4% of DM subjects.^58^ In another model, patients’ socio-demographic variables, general health condition, and periodontal status, determined by the percentage of teeth having mobility and gingival recession, were assessed as potential predictors. The results showed acceptable calibration, discrimination, and clinical values for the model.^59^ These studies showed that periodontal measurement could help identify individuals with diabetes.

### Biological mechanisms by which diabetes may induce periodontal or peri-implant diseases

Several mechanisms have been suggested to explain the increased susceptibility to periodontal or peri-implant diseases among patients with DM, including increased advanced glycation end products (AGEs) formation, enhanced oxidative stress and expression of inflammatory cytokines, dysregulated function of immune cells, uncoupled resorptive and formative responses in connective tissue, and microbial dysbiosis.

#### Advanced glycation end products

The irreversible nonenzymatic glycation and glycoxidation of proteins, such as plasma proteins, lipoproteins, and intracellular proteins produce AGEs. Elevated blood glucose leads to excessive AGE accumulation in serum, cells, and tissues.^60^^,^ ^61^ AGEs binding to its receptors (RAGEs) can induce the production of reactive oxygen species (ROS), the activation of nuclear factor-kB (NF-kB) and expression of NF-kB controlled genes, and the generation of inflammatory cytokines like tumor necrosis factor-alpha (TNF-α), and interleukin-6 (IL-6).^62^ Moreover, AGEs-RAGEs interactions trigger the production of matrix metalloproteinases (MMPs) endopeptidases with a vital role in the extracellular matrix's degradation, turnover, and remodelling.^61^^,^ ^63^ These mechanisms can inhibit the development of osteoblasts, induce apoptosis of these cells, and lead to impaired bone healing.^40^^,^ ^60^ In a study on patients with diabetes, significant positive correlations were discovered between AGEs levels and peri-implant parameters such as probing depth and crestal bone loss.^64^ In 2022, a systematic review was conducted investigate effects of inflamed periodontal tissues and periodontal pathogens on modulating AGE levels in individuals with or without T2DM and its influence on the glycaemic status. Findings revealed that individuals with T2DM and periodontitis had significantly greater levels of AGE in their gingival crevicular fluid (GCF) (521.9 pg/mL) compared to healthy individuals with periodontitis (234.84 pg/mL).^65^ Results of a recent systematic review of 16 cross-sectional studies revealed that periodontitis patients with diabetes exhibit different levels of AGEs and RAGE expressions compared to periodontitis patients without diabetes.^66^ These findings could strengthen the association between systemic AGEs and periodontitis and demonstrate AGEs as a potential link between DM and PD.

#### Oxidative stress

Diabetes, through various mechanisms, leads to increased ROS production and chronic inflammation.^67^ Hyperglycaemia increases superoxides production in the mitochondria. In turn, superoxide overproduction activates several pathways that lead to diabetic complications, including polyol pathway flux, activation of the hexosamine pathway and protein kinase C, increased AGEs production and RAGEs expression, and AGEs-RAGEs interaction.^60^

Furthermore, increased oxidative stress has been shown to induce osteoblast apoptosis in periodontal tissues.^60^ In addition to these pathogenic mechanisms occurring in patients with diabetes, the subgingival microbiome in periodontitis creates a unique environment in which phagocytes like polymorphonuclear neutrophils (PMNs) release higher amounts of ROS and exacerbate the periodontal destruction.^61^

#### Neutrophil dysfunction

The role of PMN cells in PD and diabetes has been well investigated.^68^ The persistence of neutrophils or excessive activation in the periodontal tissue can promote a chronic inflammatory state, contributing to tissue damage and periodontitis.^69^ It has been shown that in individuals with T2DM, peripheral blood neutrophils show delayed spontaneous apoptosis.^70^ Inhibition of PMN apoptosis, mediated by AGEs-RAGEs interaction, may increase retention of PMNs in the periodontal tissue, causing more tissue destruction by continuous release of ROS and MMPs.^70^^,^ ^71^ Studies have shown that PMN cell counts significantly correlate with diabetes control^72^ and the severity of periodontitis.^73^

Furthermore, it has been hypothesised that neutrophil extracellular trap (NET) formation might have a role in periodontal destruction in T2DM patients. NETs are web-like structures formed by neutrophils as a defense mechanism. On the other hand, excess production of NETs, as seen in inflammatory systemic diseases like DM, may activate immune responses and lead to periodontal tissue damage.^68^^,^ ^74^ Finally, in DM patients, neutrophils often show impaired chemotaxis and phagocytosis, reducing their ability for bacterial killing and leading to increased periodontal destruction.^75^^,^ ^76^

#### Disrupted macrophage homeostasis

It has become clear that macrophages play a central role in the pathogenesis of diabetic complications, including periodontitis.^77^ According to that, there are 2 phenotypes of macrophages: M1 macrophages, which produce inflammatory cytokines and induce osteoclast activation, and M2 macrophages, which are associated with inflammation resolution and tissue repair.^78^ Results of a study on periodontitis patients with or without T2DM showed that in periodontally affected sites, the M1/M2 macrophage ratio was significantly higher. Besides, the M1 macrophage abundance was higher in T2DM patients' healthy sites than in non-DM patients' healthy sites.^79^ Another study suggested complement 3 (C3)-mediated polarisation of macrophages as a possible mechanism for periodontal destruction. First, it was shown that T2DM patients had elevated C3 in their blood and GCF and also had worse periodontal conditions compared to individuals without diabetes.^78^ Results of a study suggest that hyperglycaemia induces glucose transporter protein type 1 (GLUT1)-mediated macrophage senescence and inflammation. These 2 responses, individually and by interacting with each other, contribute to the susceptibility and severity of PD and may have essential roles in diabetes-related PD.^80^

#### Proinflammatory factors

It has been proposed that increased systemic levels of proinflammatory cytokines associated with diabetes may affect the immune reactions in periodontal tissues. On the other hand, in periodontitis conditions, proinflammatory cytokines generated locally in periodontal tissues have the potential to permeate the bloodstream, affect distant tissues and organs, and lead to insulin resistance and beta cell destruction.^61^

The most investigated inflammatory cytokines in the studies are IL-1β, IL-6, and TNF-α. TNF-α, mainly produced by macrophages, can inhibit osteoblast differentiation and formation, leading to reduced bone formation. Likewise, TNF-α can induce bone resorption by altering osteocyte metabolism function.^81^ IL-1β, which is mainly secreted by macrophages, induces the production of other inflammatory mediators (eg, prostaglandin E2 (PGE2) and IL-6). IL-6, expressed by most immune system cells, is the most critical mediator for the induction of acute-phase proteins (eg, CRP) and is vital for osteoclast formation and MMP production.^61^ TNF-α and IL-6 have been associated with impaired intracellular insulin signalling, potentially leading to insulin resistance and further diabetes complications.^71^^,^ ^82^

Many studies have shown that the levels of these cytokines in saliva, GCF, or serum of periodontitis patients with diabetes are higher compared to systematically healthy patients.^83^, ^84^, ^85^, ^86^ One systematic review reported that even patients with diabetes with better glycaemic control might still have higher GCF levels of IL-1β than healthy individuals.^87^ Interleukin-8 (IL-8), a proinflammatory cytokine that has a role in the metabolism of ROS and osteoclastogenesis,^88^ has been reported to be elevated in periodontitis patients with T1DM and T2DM compared to non-DM patients.^84^^,^ ^89^ It might have a role in these diseases' pathogenesis and association.

Interleukin-17 (IL-17) is another cytokine acknowledged as a contributor to diabetes-enhanced periodontitis.^90^ Studies have demonstrated that T2DM enhanced IL-17 levels in periodontitis patients.^91^^,^ ^92^ In addition, higher IL-17 levels have been reported to be correlated with worse periodontal parameters in T2DM patients.^93^ Diabetes-enhanced IL-17 stimulates the expression of other inflammatory mediators such as IL-6, IL-8, PGE2, TNF-α, and RANKL and can indirectly enhance osteoclastogenesis, thereby increasing periodontal tissue damage.^94^^,^^95^

Regarding dental implants, a systematic review has reported that the central cytokines associated with peri-implantitis were IL-1β, IL-6, IL-17, TNF-α, RANK, and RANKL.^96^ It has also been shown that among patients with peri-implantitis, salivary IL-1β, and IL-6 levels were significantly higher in those with diabetes than in those without diabetes.^97^ However, one study found no significant differences in GCF and peri-implant crevicular fluid IL-1 and TNF- levels around implants between well-controlled patients with diabetes and healthy controls.^98^

#### Uncoupled resorptive and formative responses in connective tissue

The increased levels of periodontal tissue destruction in patients with diabetes may be connected to the alterations in connective tissue metabolism, leading to uncoupled resorptive and formative processes and reduced healing capacity.^61^ It is suggested that MMPs play a role in the association between DM and PD. Results of a meta-analysis of eight studies on the possible role of MMP-8 in the pathogenesis of PD in patients with diabetes revealed that there are higher levels of MMP-8 in patients with PD compared to those without PD.^99^ Higher GCF MMP-2 levels were reported in periodontitis patients with DM compared to non-DM individuals.^86^

In a recent case-control study, Arshad et al. assessed the relation between levels of MMP-9 and T2DM in chronic periodontitis patients. Authors reported that the levels of MMP-9 in DM patients with chronic periodontitis were nearly doubled (156.95 ± 29.80 ng/mL) compared to the values in the control group (74.96 ± 6.32 ng/mL) (*P* < .001). Moreover, periodontal parameters were significantly more severe in DM individuals and chronic periodontitis in comparison to the control group.^100^

Diabetes induces apoptosis of matrix-producing cells, including fibroblasts in gingiva and periodontal ligament (PDL), by activating the caspase-3 signalling pathway.^101^, ^102^, ^103^ Diabetes also contributes to bone loss by augmenting inflammation and uncoupling the bone resorption and formation processes in periodontal tissue. The main signalling pathway controlling osteoclastic resorptive and osteoblastic formative functions is RANK/RANKL/OPG.^104^^,^ ^105^ Under inflammatory conditions like diabetes and periodontitis, cytokines such as TNF-α, IL-1, and PGE2 enhance osteoclast formation and activity via RANK signalling in osteoclast progenitors.^81^^,^ ^106^ Higher amounts of proinflammatory cytokines and RANKL were seen in the GCF of T2DM patients with chronic periodontitis compared to non-DM patients.^91^ Moreover, through enhanced inflammation, hyperglycaemia causes a decrease in bone formation and a reduction in the mechanical characteristics of newly created bone, reducing the repair capacities of periodontal bone.^60^^,^ ^75^

#### Microbiome alterations

Recently, more attention has been drawn to the possible microbial changes due to DM and hyperglycaemic state that could have a role in the worsening of periodontal condition.^107^ However, the literature on this issue still represents conflicting results.

A systematic review evaluated the literature on the subgingival microbial profile of patients with chronic periodontitis. It was concluded that in periodontitis patients with T2DM, periodontal pathogens such as *Tannerella forthysia* (T. forsythia), *Aggregatibacter actinomycetemcomitans* (A. actinomycetemcomitans), and *Porphyromonas gingivalis* (P. gingivalis) were less frequently found compared to non-DM patients. The strength of evidence was the highest for T. forsythia.^108^ This finding contrasts a study that reported a higher abundance of T. forsythia in the subgingival microbiome of DM patients compared to controls.^109^ In a survey of the same issue/matter, it was reported that when comparing periodontitis patients with and without DM, a lower abundance of Veillonellaceae and a higher abundance of Neisseriaceae were seen in DM subjects compared to patients without diabetes.^110^

Data from one study showed a higher diversity of classic periodontopathogens in the subgingival microbiome of periodontitis patients with T2DM than those without DM.^109^ Similarly, in a study of children with T1DM, higher HbA1c levels independently increased diversity in oral (gingival but not buccal) microbiota.^111^ Conversely, another study reported that an increase in saliva microbial diversity was observed in periodontitis patients with or without T2DM, indicating that this increase in diversity may not be related to diabetes.^112^ One cohort study on T2DM and systematically healthy patients showed that the difference in subgingival microbiome between healthy and the periodontitis state was less pronounced in T2DM patients compared to healthy subjects, suggesting that small shifts toward dysbiosis in the microbiome could trigger periodontitis in these patients, which could be a result of compromised host metabolic and immune regulation. The study also reported that in the healthy state, the subgingival microbiome of T2DM patients was more pathogenic, suggesting that they are at an increased risk of developing periodontitis compared to non-DM subjects. T2DM patients, on the other hand, had lower relative abundances of orange complex and red complex species in the resolved state than non-DM patients, indicating that T2DM patients are less tolerant of the presence of periodontal pathogens and have lower pathogen abundances to keep the periodontium in a clinically resolved state.^113^

Recently, a case-control study by Yu et al. showed that DM impact the advancement of periodontitis by enhancing the bacterial network within the gingival tissue.^114^ Further in 2023, another case-control study evaluated the diversity and prevalency of periodontal pathogens found in the subgingival plaque of individuals with severe chronic periodontitis, with and without diabetes. The findings of this study demonstrated a greater bacterial burden in the DM group, *T*. forsythia (*P* < .037) and *T*. denticola (*P* < .003), compared to the non-DM group.^115^

Considering the inconsistency of data among the studies on the influence of microbiome alterations by DM, it is difficult to determine whether diabetes can independently alter the oral microbial composition and increase its pathogenicity to cause more susceptibility to PD. Further studies are required to evaluate the possible role of microbial change in the relationship between these two diseases.

## Conclusion and future perspective

The results of the reviewed studies demonstrated that DM and periodontal/peri-implant disease are bidirectionally associated, and this association seems to be causal. However, this causality appears more robust when DM is the exposure. Additionally, it was shown that DM is a significant risk factor for periodontal/peri-implant disease. In addition to inflammation, which is the critical player in the connection between these diseases, other factors, such as microbiome alterations, may have a role (Figure). The reviewed studies suggest that the presence of chronic hyperglycaemia is a more significant risk factor for periodontal and peri-implant diseases than a specific type of diabetes. Among DM patients, those with poor glycaemic control have a higher prevalence and severity of periodontal/peri-implant disease, as evidenced by worse periodontal parameters and a greater risk of tooth loss.

**Fig. 1:**
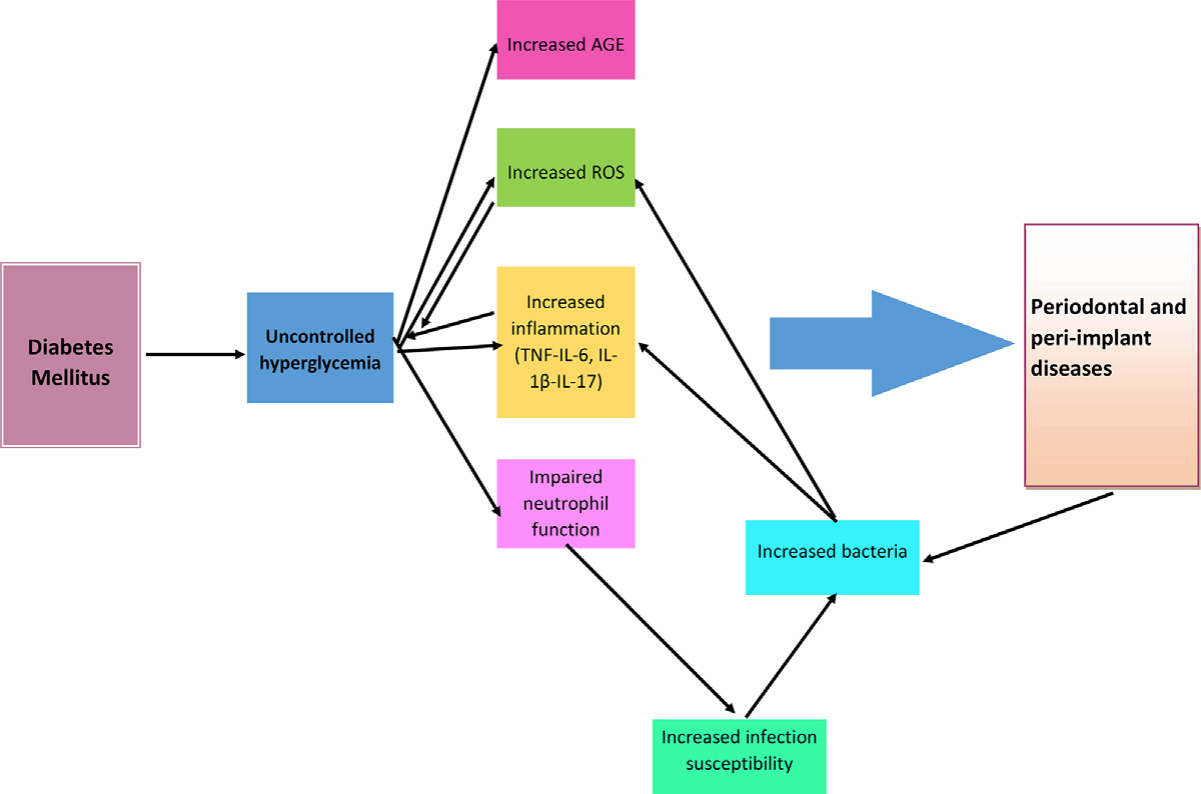
Schematic summary of pathways depicting a bidirectional relationship between diabetes mellitus and periodontal/peri implant diseases and its potential related mechanisms.

Although the majority of studies report a higher prevalence and risk of periodontal and peri-implant diseases in DM patients, this risk varies across studies, and considerable heterogeneity is observed. This heterogeneity is due to differences in diagnostic criteria and classification methods for these diseases based on population surveillance, clinical assessment, or self-reported data. In addition, definitions of periodontal and peri-implant diseases, as well as the cut-off values defining diabetic status, have changed considerably over time. Moreover, there is variation among studies regarding the consideration of confounding factors such as age, sex, body mass index, hypertension, smoking status, oral hygiene, etc. More studies with a valid assessment of these conditions and proper adjustment of measured confounders are warranted.

When managing DM patients, there is a lack of interprofessional patient care between medical and dental practitioners.^116^ Both dentists and physicians should be aware of the link between DM and periodontal/peri-implant diseases. Medical doctors with a broad understanding of basic oral health can aid in the early diagnosis of PD. Dentists, on the other hand, should be alert that periodontitis might be a sign of undetected diabetes and poor metabolic control. Dental professionals should be vigilant in preventing and treating PD, not only to preserve dentition but also to potentially improve metabolic control^117^^,^ ^118^ and decrease micro- and macrovascular complications in DM patients.^119^, ^120^, ^121^

However, as we reflect on the wealth of existing literature, it becomes evident that there are notable gaps in our mechanistic understanding, largely attributed to the limitations in current evaluation methods. The complexities of the diabetic-periodontal relationship demand more reliable approaches and comprehensive methodologies to pave the way for the identification of previously undiscovered links, shedding light on the intricate molecular and cellular mechanisms that define the relationship between diabetes mellitus and periodontitis. Future research endeavours should strive to overcome these methodological challenges, employing advanced techniques and interdisciplinary collaborations to dissect the intricate pathways linking diabetes and periodontal disease. Additionally, deeper understanding of the intricate mechanistic connections between diabetes mellitus and periodontitis holds the potential to unveil novel insights into the directional association between these two conditions. One area deserving research is to explore the impact of novel-antidiabetic medications, which have been shown to possess diverse pleiotropic actions,^122^, ^123^, ^124^, ^125^, ^126^, ^127^, ^128^ on the incidence and severity of periodontal disease. Moreover, in the future longitudinal prospective investigations, it is crucial to tackle methodological challenges, including the absence of data pertaining to periodontal damage and the constraints imposed by small sample sizes. Subsequent research endeavours might also involve assessing the cluster effect of prevalent risk variables, aiming to elucidate the combined impact of blood glucose levels and these risk factors on the initiation and progression of periodontitis.

## Conflict of interest

None declared.
